# A cross‐sectional study of the association between effort‐reward imbalance and psychologic distress among Japanese dietitians

**DOI:** 10.1002/1348-9585.12285

**Published:** 2021-10-22

**Authors:** Kozue Yaginuma‐Sakurai, Chotoku Saito, Yoshiko Kasahara, Kanami Tsuno, Kouichi Yoshimasu, Nozomi Tatsuta, Miki Goto, Kunihiko Nakai

**Affiliations:** ^1^ Department of Human Health and Nutrition College of Human Health and Nutrition Shokei Gakuin University Natori Miyagi Japan; ^2^ Graduate School of Comprehensive Human Sciences Master’s Course in Nutritional Science Shokei Gakuin University Natori Miyagi Japan; ^3^ Department of Nutrition Faculty of Health Science Aomori University of Health and Welfare Aomori Aomori Japan; ^4^ Department of Food and Health Sciences Faculty of Health and Human Development The University of Nagano Nagano Nagano Japan; ^5^ School of Health Innovation Kanagawa University of Human Services Kawasaki Kanagawa Japan; ^6^ Department of Psychological and Behavioral Sciences School of Human Sciences Kobe College Nishinomiya Hyogo Japan; ^7^ Department of Development and Environmental Medicine Tohoku University Graduate School of Medicine Sendai Miyagi Japan; ^8^ School of Sport and Health Science Tokai Gakuen University Miyoshi Aichi Japan

**Keywords:** coworker support, dietitians, effort‐reward imbalance, psychologic distress, supervisor support

## Abstract

**Objectives:**

This study aims to clarify dietitians’ effort‐reward imbalance (ERI) and examine its association with psychologic distress.

**Methods:**

A cross‐sectional survey was conducted. A total of 3593 questionnaires were distributed to dietitians in about 110 organizations and 1890 responses were received (response rate 52.6%). Hence, a total of 1743 valid questionnaires were used in the analysis. Effort‐reward (ER) ratio was measured by a subscale of the ERI Questionnaire, and psychologic distress was measured by the Kessler Psychological Distress Scale (K6). The association between the ER ratio and psychologic distress was analyzed by multiple logistic regression analysis with covariates.

**Results:**

The mean ER ratio was 0.83 (SD = 0.53) and ERI (ER ratio >1) prevalence was 26.3%. The mean K6 score was 7.1 (5.3), and psychologic distress (K6 score ≥5) prevalence was 62.4%. The increased psychologic distress was associated with a higher ER ratio, less support from supervisors and coworkers, and lower age and household income. ERI was significantly associated with psychologic distress, even after being adjusted for covariates.

**Conclusions:**

Dietitians experience high stress, as shown by their high ER ratio and K6 scores. Their ERI was greatly associated with psychologic distress.

## 1 INTRODUCTION

Due to the rapid aging of the population and a declining birthrate, there have been many problems related to nutrition and dietary habits such as disordered eating habits and increased lifestyle‐related diseases. These have become urgent issues to be resolved in Japan. Accordingly, the work environment of dietitians has changed significantly. They need to improve the quality of their work now more than ever.

A medical team consisting of dietitians and other medical professionals called the Nutrition Support Team (NST) was introduced in the clinical field. This was to accommodate the expansion of team‐based medicine. In addition, medical service fees were revised, with additional fees added for nutritional management.^1^ Due to the increased integration of nutritional management in clinical settings, dietitians in the clinical fields have acquired additional duties (such as increased interaction with other medical professionals and patients). In the school health field, the prevalence of food allergies has been increasing yearly.^2^ It demands careful attention for managing school lunches for children and infants with food allergies.^3^ In addition, dietary education has become critical in various fields. Nutrition guidance is now needed for children and people of various ages and positions, expanding the dietitians’ activities as dietary education providers.

The social need for nutrition management specialists has increased, leading to changes in their work duties. Consequently, while dietitians can maintain a high level of professional efficacy, the increased work and complicated interpersonal relationships have led to increased stress. Various surveys have been conducted for occupational stress.^4^, ^5^, ^6^, ^7^, ^8^, ^9^, ^10^, ^11^, ^12^ However, only a few studies have explored this for dietitians.^13^, ^14^, ^15^, ^16^ Most of these surveys have been conducted with dietitians working in hospitals or social welfare facilities.^13^, ^14^, ^15^, ^16^ However, dietitians work in various settings including hospitals, social welfare facilities, nursery schools, elementary schools, junior high schools, food companies, research and educational institutions, and government facilities. Thus, conducting surveys in different settings may clarify their stress status since their tasks vary according to the workplace.

Different models of work stress have illustrated the mechanism that underpins occupational health.^17^, ^18^, ^19^, ^20^ Among these is the effort‐reward imbalance (ERI) model, which predicts health conditions in occupation life with two axes, “effort” and “reward.” This model appropriately describes the stressful professional work environment that is readily influenced by client rewards.^20^, ^21^ Previous studies indicated that depression, sick leaves, burnout, and psychologic distress were strongly related to ERI in various workplaces.^9^, ^10^, ^21^, ^22^, ^23^ However, no previous studies to our knowledge have researched ERI among dietitians.

Therefore, we conducted a cross‐sectional survey of dietitians in various workplaces to assess their ERI. We also examined the association between ERI and psychologic distress in dietitians. Previous studies have already proven this association exists for other fields such as clerical staff members and local government employees.^9^, ^10^ Further, the results of this study clarify dietitians’ stress conditions and their causes, which have not been identified by previous studies.

## 2 METHODS

### 2.1 Participants

A cross‐sectional study was carried out in Japan among dietitians who belonged to or worked at: (1) the Prefectural Dietic Associations of 13 prefectures, (2) two contracted food service companies in the Tōhoku region, and (3) municipal offices, childcare facilities (nursery schools and certified *kodomo*‐*en*), elementary schools, and junior high schools in the Miyagi prefecture. Registered dietitians have a more advanced national qualification and can provide guidance to patients in medical institutions compared with dietitians, but their duties may be almost the same in some workplaces. Therefore, both registered dietitians and dietitians were collectively included as dietitians in this study. The survey period was from May 2018 to November 2019.

The survey was conducted during meetings and workshops conducted by the organizations mentioned and by postal mail. We distributed self‐administered anonymous questionnaires after explaining the purpose of the study to all candidates. The ones distributed by postal mail were sent through candidates’ organizations that they mailed back to us upon completion. In addition, careful consideration was given to avoid overlap between participants at the meetings, workshops, and those who participated by mail. In total, we distributed 3593 questionnaires and received 1890 responses (response rate = 52.6%). Of these 147 questionnaires, there were missing data for the study variables (gender, age, certification, workplace, supervisor support, coworker support, effort‐reward [ER] ratio, and psychologic distress). These were not included in the analyses. An “unknown” category was established for questionnaires with household income (*n* = 259) and length of employment at current workplace (*n* = 37) values not provided and these variables were then included in the analysis. A total of 1743 responses were eventually analyzed in this survey.

The study was approved by the Research Ethics Committee of Shokei Gakuin University (Approval Number: 017‐022). The study participants were fully informed of the purpose of the study and answered the questionnaire anonymously. Their participation was entirely voluntary, and their responses were returned directly to the researchers.

### 2.2 Measures

The details have been described elsewhere.^4^ In short, the ERI was measured using a subscale of the ERI Questionnaire,^20^, ^24^ psychologic distress using the Kessler Psychological Distress Scale (K6),^25^, ^26^, ^27^ and worksite social support from supervisors and coworkers using a subscale of the Job Content Questionnaire (JCQ).^18^, ^28^ In the current study, the internal reliability of these scales was high, with Cronbach's α of .905 (effort of ERI), .897 (reward of ERI), .895 (psychologic distress of K6), .909 (supervisor support of JCQ), and .845 (coworker support of JCQ). In addition, we also established gender, age, household income, certification (whether they were registered dietitians or dietitians), and workplace (hospital, social welfare facilities, nursery center, school or school lunch center, municipal facilities, and others) as covariates.

### 2.3 Statistical analysis

Using a χ^2^ test, we analyzed the differences between participants who experienced psychologic distress and did not (K6 ≥5). Furthermore, residue analysis was carried out in tests with three or more groups. The association between ER ratio and psychologic distress was analyzed by using univariate and multivariate logistic regression to calculate the odds ratios (ORs) and 95% confidence intervals (CIs). We adjusted covariates for gender, age, household income, certification, workplace, supervisor support, and coworker support in the multivariate logistic regression analysis. In addition, we checked for multicollinearity using Spearman's rank correlation coefficient between ER ratio and the given covariates. The significance level was set at *p* < .05 (two‐sided) for all statistical analyses. All statistical analyses were carried out with software package of JMP, version 14.3 (SAS Institute Inc.).

## 3 RESULTS

**FIGURE 1:**
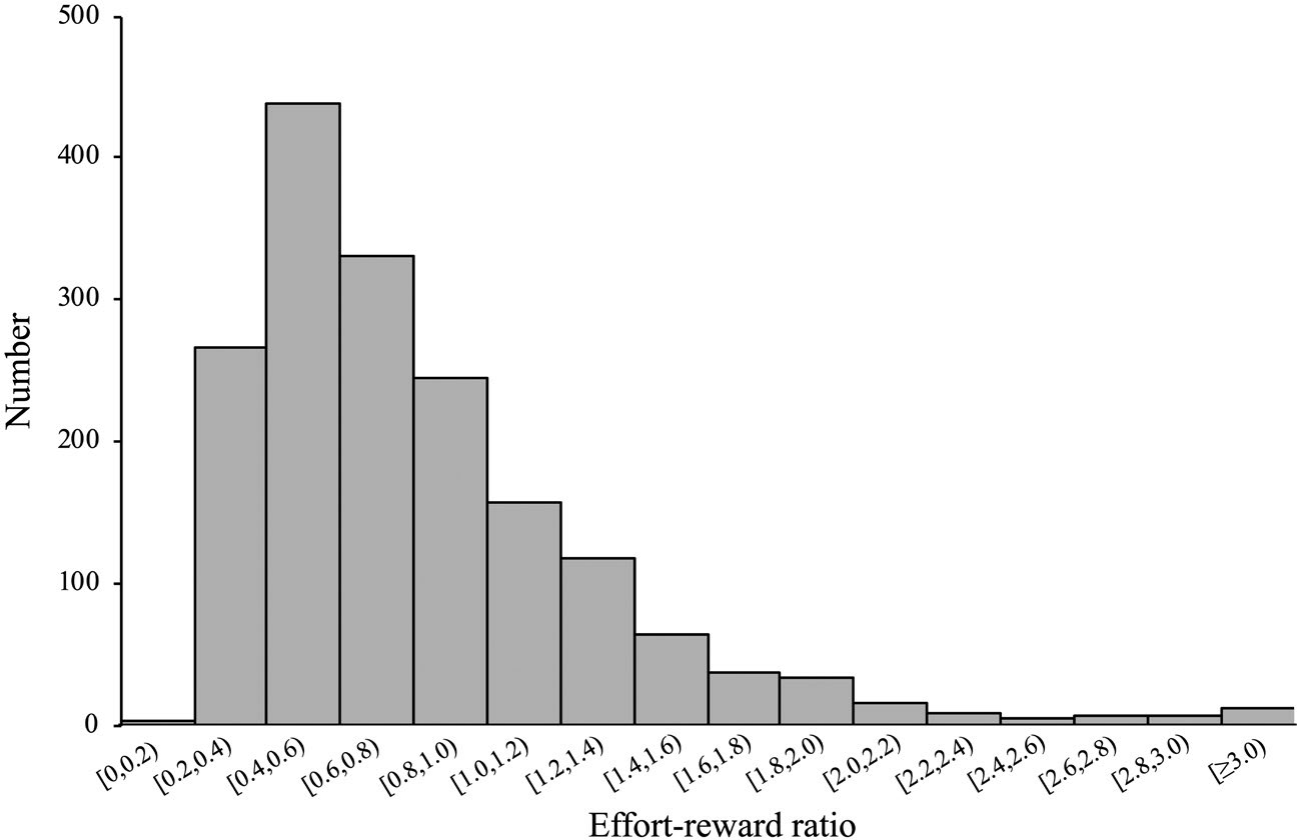
Distribution of the effort‐reward ratio for the participants (*n* = 1743)

The analysis of the participants’ characteristics follows the presence or absence of psychologic distress, as shown in Table 1. The overall mean K6 score was 7.1 (SD = 5.3), and there was a 62.4% prevalence of psychologic distress (K6 score ≥5). Participants with psychologic distress tended to be younger (*p* < .001), had a lower household income (*p* < .001), were not licensed dietitians (*p* < .001), worked at social welfare facilities (*p* = .003), and received lower support from supervisors (*p* < .001) and coworkers (*p* < .001). The overall mean scores of supervisor and coworker support were 11.6 (2.5) and 11.9 (2.1), respectively. For the ERI factors, the mean score of effort was 17.1 (6.3) and that of reward was 42.5 (9.2). Thus, the mean ER ratio was 0.83 (0.53). The distribution of the ER ratio is shown in Figure 1.

**TABLE 1:** Characteristics of the participants by psychologic distress (*n* = 1743)

	K6 <5 (*n* = 655)		K6 ≥5 (*n* = 1088)		χ^2^	*P* ^a^
	*n* (%)	Adjusted residual	*n* (%)	Adjusted residual		
Gender						
Male	39 (6.0)		71 (6.5)		0.226	.635
Female	616 (94.0)		1017 (93.5)			
Age (years)						
<30	149 (22.7)	−3.3	327 (30.1)	3.3	21.03	**<.001**
30–39	138 (21.1)	−1.6	265 (24.4)	1.6		
40–49	137 (20.9)	1.0	207 (19.0)	−1.0		
≥50	231 (35.3)	3.8	289 (26.6)	−3.8		
Household income (Japanese yen/year)						
<5 million	204 (31.1)	−5.3	478 (43.9)	5.3	38.32	**<.001**
5–10 million	249 (38.0)	2.0	363 (33.4)	−2.0		
≥10 million	100 (15.3)	4.5	90 (8.3)	−4.5		
Unknown	102 (15.6)	0.6	157 (14.4)	−0.6		
Certification						
Registered dietitian	528 (80.6)		763 (70.1)		23.39	**<.001**
Dietitian	127 (19.4)		325 (29.9)			
Workplace						
Hospital	265 (40.5)	0.3	433 (39.8)	−0.3	17.90	.**003**
Social welfare facilities	107 (16.3)	−3.3	250 (23.0)	3.3		
Childcare facilities	44 (6.7)	−0.8	85 (7.8)	0.8		
School or school lunch center	56 (8.5)	0.1	91 (8.4)	−0.1		
Municipal facilities	81 (12.4)	2.3	97 (8.9)	−2.3		
Others	102 (15.6)	2.0	132 (12.1)	−2.0		
Length of employment at current workplace (years)						
<1	76 (11.6)	−1.6	155 (14.2)	1.6	8.52	.074
1–5	225 (34.4)	−1.1	402 (36.9)	1.1		
5–10	115 (17.6)	−0.4	199 (18.3)	0.4		
≥10	222 (33.9)	2.2	315 (29.0)	−2.2		
Unknown	17 (2.6)	1.5	17 (1.6)	−1.5		
Supervisor support (JCQ)						
High (≥median)	495 (75.6)		599 (55.1)		73.65	**<.001**
Low (<median)	160 (24.4)		489 (44.9)			
Coworker support (JCQ)						
High (≥median)	505 (77.1)		633 (58.2)		64.58	**<.001**
Low (<median)	150 (22.9)		455 (41.8)			
Effort (ERIQ)						
High (≥median)	214 (32.7)		746 (68.6)		212.9	<.001
Low (<median)	441 (67.3)		342 (31.4)			
Reward (ERIQ)						
High (≥median)	504 (76.9)		415 (38.1)		247.0	<.001
Low (<median)	151 (23.1)		673 (61.9)			
Effort‐reward ratio (ERIQ)						
≤1	610 (93.1)		674 (61.9)		204.9	**<.001**
>1	45 (6.9)		414 (38.1)			

Abbreviations: ERIQ, Effort‐Reward Imbalance Model Questionnaire; JCQ, Job Content Questionnaire; K6, Kessler Psychologic Distress Scale.

^a^ The differences were tested by χ^2^ test, and residue analysis was performed in tests with three or more groups.

Participants with psychologic distress had higher effort scores (*p* < .001), lower reward scores (*p* < .001), and higher ER ratios (*p* < .001). The overall prevalence of ERI (ER ratio >1) was 26.3% and for participants with psychologic distress was 38.1%. This percentage was significantly higher for those without psychologic distress, 6.9% (*p* < .001).

Table 2 shows the results of logistic regression analysis on the association of ERI with psychologic distress after adjusting gender, age, household income, certification, workplace, supervisor support, and coworker support. Psychologic distress was significantly associated with an ER ratio of >1 (6.70, 95% CI, 4.76–9.41), age group of <30 (1.74, 95% CI, 1.28–2.36) and 30–39 (1.40, 95% CI, 1.03–1.90), household income of <5 million (1.88, 95% CI, 1.31–2.70), low supervisor support (1.54, 95% CI, 1.20–1.97), and low coworker support (1.68, 95% CI, 1.31–2.17). When the same analysis was performed with the addition of effort score and reward score except for the ER ratio, psychologic distress was significantly associated with high effort (3.11, 95% CI, 2.46–3.93) and low reward (3.40, 95% CI, 2.65–4.35). Conversely, gender, age, certification, and workplace were not significantly associated with psychologic distress. The highest value of Spearman's rank correlation coefficient was 0.472 between the supervisor and coworker support. Thus, we found that this model had no evidence of multicollinearity.

**TABLE 2:** Univariable and multivariable logistic regression analyses regarding covariates associated with psychologic distress (*n* = 1743)

	Univariable		Multivariable	
	OR (95% CI)	*P* ^a^	OR (95% CI)	*P* ^a^
Effort‐reward ratio (ERIQ)				
≤1	Reference		Reference	
>1	8.33 (6.01–11.5)	**<.001**	6.70 (4.76–9.41)	**<.001**
Gender				
Male	Reference		Reference	
Female	0.91 (0.61–1.36)	.635	0.95 (0.61–1.47)	.815
Age group (years)				
<30	1.75 (1.35–2.27)	**<.001**	1.74 (1.28–2.36)	**<.001**
30–39	1.53 (1.17–2.01)	.**002**	1.40 (1.03–1.90)	.**030**
40–49	1.21 (0.92–1.59)	.181	1.12 (0.82–1.52)	.482
≥50	Reference		Reference	
Household income (Japanese Yen/year)				
<5 million	2.60 (1.87–3.62)	**<.001**	1.88 (1.31–2.70)	**<.001**
5–10 million	1.62 (1.17–2.25)	.**004**	1.20 (0.84–1.72)	.306
≥10 million	Reference		Reference	
Unknown	1.71 (1.17–2.50)	.**005**	1.02 (0.67–1.56)	.931
Certification				
Registered dietitian	Reference		Reference	
Dietitian	1.77 (1.40–2.24)	**<.001**	1.26 (0.96–1.66)	.094
Workplace				
Hospital	Reference		Reference	
Social welfare facilities	1.43 (1.09–1.88)	.**010**	1.18 (0.87–1.60)	.297
Childcare facilities	1.18 (0.80–1.75)	.406	1.07 (0.69–1.67)	.748
School or school lunch center	0.99 (0.69–1.43)	.977	1.09 (0.72–1.65)	.689
Municipal facilities	0.73 (0.53–1.02)	.067	1.09 (0.76–1.56)	.642
Others	0.79 (0.59–1.07)	.128	0.99 (0.71–1.39)	.977
Supervisor support (JCQ)				
High (≥median)	Reference		Reference	
Low (<median)	2.53 (2.04–3.13)	**<.001**	1.54 (1.20–1.97)	**<.001**
Coworker support (JCQ)				
High (≥median)	Reference		Reference	
Low (<median)	2.42 (1.95–3.01)	**<.001**	1.68 (1.31–2.17)	**<.001**

Abbreviations: CI, confidence interval; ERIQ, Effort‐Reward Imbalance Model Questionnaire; JCQ, Job Content Questionnaire; OR, odds ratio.

^a^ The differences were tested by multivariate logistic regression analysis adjusted for gender, age group, household income, certification, workplace, supervisor support, and coworker support.

## 4 DISCUSSION

This study clarified the state of the ERI among dietitians and examined its association with psychologic distress. The mean ER ratio was 0.83, and the prevalence of ERI was 26.3% among the study participants. A significant association was found between ERI and psychologic distress. As previously mentioned, there have been few surveys on work‐related stress among dietitians based on the ERI model. This study is the first to research ERI among dietitians. It is expected that the results of this study could be a basis to review the working system and salaries of dietitians.

In previous surveys, the mean ER ratios for other occupations were reported to be 0.5 for 1000 female office employees,^29^ 0.7 for 2208 female specialists,^29^ 0.8 for 348 nurses,^7^ 0.93 for 1210 childcare workers,^4^ and 1.38 for 342 eldercare workers^8^ in Japan. Compared with other occupations, we found the mean ER ratio of dietitians to be higher than that of female office employees and specialists,^29^ lower than that of childcare^4^ and eldercare workers,^8^ and comparable to nurses.^7^ However, this comparison was not adjusted for gender, age, and other covariates. Dietitians’ ER ratio exhibited greater effort values (regarding work content and volume) than office employees, who were in the best ERI status compared with other occupations.^29^ They also exhibited greater reward values (including job satisfaction) than eldercare workers (who were in the poorest ERI status).^8^ A previous survey with hospital dietitians reported that although their quantitative and qualitative burdens of work and interpersonal stress were high, they felt capable of working in environments that required advanced skills and knowledge. Further, they reported high work satisfaction.^15^ Our study reported similar findings in terms of ERI, which characterizes dietitians’ professional life. Furthermore, the study examined whether effort or reward was a more important factor in psychologic distress and found that the relationship was comparable.

In the current study, the average K6 score was 7.1. In previous studies, K6 scores were reported to be 5.2 for 2191 local government employees,^5^ 5.6 for 60 female employees in the manufacturing industry,^11^ 6.2 for 348 female nurses,^7^ 7.0 for 1210 childcare workers,^4^ and 7.7 for 789 nurses.^6^ Thus, compared with these professions, the dietitians appear to belong to the group of highly stressed occupations.

We also surveyed work‐related social support as a covariate of psychologic distress and found that supervisor support was 11.8 and coworker support was 11.9. These values are comparable with those reported for childcare workers (supervisor support: 11.8; coworker support: 12.1)^4^ and slightly higher than female government workers (supervisor support: 10.5; coworker support: 11.0)^12^ in previous studies. Additionally, in many cases, there is only one dietitian assigned to a workplace or the dietitians’ supervisors are from a different profession.^30^ In such cases, dietitians’ stress can be higher, as they may lack colleagues to confide in, or their supervisor may lack the expertise to assess their work correctly.^30^ In this study, a strong relationship between psychologic distress and ERI was observed after adjusting for supervisor and coworker support, while a significant effect of supervisor and coworker support on psychologic distress was also observed. Therefore, it is crucial to develop appropriate working environments for dietitians with appropriate supervisors and coworkers’ support, even if they are from other professions.

Our multiple logistic regression analysis results revealed that ER ratio was significantly associated with dietitians’ psychologic distress even after adjusting for covariates. Thus, our study confirms the positive association between ER ratio and psychologic distress reported by the previous studies.^9^, ^10^ We believe that an improvement of the dietitians’ ERI may directly reduce their psychologic distress.

One way to improve the ERI of dietitians would be to increase their pay. According to the 2020 Basic Survey on Wage Structure by the Ministry of Health, Labour and Welfare,^31^ the average salary of dietitians is lower than that of nurses, pharmacists, and clinical laboratory technicians. In recent years, The Japan Ministry of Health, Labour, and Welfare has been promoting salary improvements for caregivers and childcare workers.^32^, ^33^ However, there has been little improvement in dietitians’ salaries, even though the demand for nutrition specialists has been increasing. The dietitians’ salaries should be increased for a better ERI.

Dietitians’ ERI could also be linked to their social status. Compared with other healthcare professionals, dietitians’ roles and work seem to be poorly understood,^30^ and the evaluation of their expertise is often insufficient. These factors could also contribute to dietitians’ low reward ratio. Therefore, to improve dietitians’ ERI, it might be necessary to improve the understanding of their role in the workplace and society and how their expertise is assessed. In addition, increasing psychologic rewards may also help improve dietitians’ work environment. For example, we reported that work engagement and job satisfaction were higher among childcare workers than nurses and other professions.^4^ Future research should examine dietitians’ work engagement.

The following limitations were included in this study. First, due to the cross‐sectional design, it was not possible to determine the causal relationships. Therefore, a longitudinal study will be needed to examine the potential causal relationships. Second, a self‐assessment method was used. Consequently, all variables were only measured using subjective indicators, which may have resulted in response bias. Objective indicators such as physiologic or biochemical indicators will be required to evaluate stress in future studies. Third, the response rate for our study was somewhat low at 52.6%, which may have caused a selection bias. In particular, the response rate for the postal mail method was low, so self‐selection bias was a concern. Fourth, overcommitment has not been investigated.

In conclusion, this study showed that dietitians are highly stressed despite the above limitations, and their ERI was significantly associated with psychologic distress.

## CONFLICT OF INTEREST

The authors declare no conflicts of interest for this article.

## AUTHOR CONTRIBUTIONS

K.Y.S. conducted the study, analyzed and interpreted data, and draft and critically revised the article. C.S. and Y.K. contacted each organization to which dietitians belong to ask for their cooperation in the survey. K.T. and M.G. revised the questionnaire. K.T., K.Y., N.T., and K.N. interpreted the data and critically revised the article. All authors read and approved the final manuscript.

## APPROVAL OF THE STUDY PROTOCOL

The study was approved by the Research Ethics Committees of the Shokei Gakuin University (Approval Number: 017‐022).

## INFORMED CONSENT

The study participants were fully informed of the purpose of the study and answered the questionnaire anonymously. Their participation was entirely voluntary, and their responses were returned directly to the researchers.

## REGISTRY AND THE REGISTRATION NO. OF THE STUDY/TRIAL

Not applicable.

## ANIMAL STUDIES

Not applicable.

## Data Availability

The data that support the findings of this study are available from the corresponding author upon reasonable request.
